# Gender Moderates Results of a Randomized Clinical Trial for the Khanya Intervention for Substance Use and ART Adherence in HIV Care in South Africa

**DOI:** 10.1007/s10461-022-03765-8

**Published:** 2022-07-27

**Authors:** Jennifer M. Belus, John A. Joska, Yosef Bronsteyn, Alexandra L. Rose, Lena S. Andersen, Kristen S. Regenauer, Bronwyn Myers, Judith A. Hahn, Catherine Orrell, Steve A. Safren, Jessica F. Magidson

**Affiliations:** 1 Department of Medicine, Swiss Tropical and Public Health Institute, Basel, Switzerland; 2 University of Basel, Basel, Switzerland; 3 Department of Psychology, University of Maryland, College Park, MD USA; 4 HIV Mental Health Research Unit, Division of Neuropsychiatry, Department of Psychiatry and Mental Health, Neuroscience Institute, University of Cape Town, Cape Town, South Africa; 5 Global Health Section, Department of Public Health, University of Copenhagen, Copenhagen, Denmark; 6 Curtin enAble Institute, Faculty of Health Sciences, Curtin University, Perth, WA Australia; 7 South African Medical Research Council, Alcohol, Tobacco, and Other Drug Research Unit, Cape Town, South Africa; 8 Division of Addiction Psychiatry, Department of Psychiatry and Mental Health, University of Cape Town, Cape Town, South Africa; 9 Department of Medicine, University of California, San Francisco, San Francisco, CA USA; 10 Desmond Tutu HIV Centre, Institute of Infectious Disease and Molecular Medicine and Department of Medicine, University of Cape Town, Cape Town, South Africa; 11 Department of Psychology, University of Miami, Miami, FL, USA

**Keywords:** ART adherence, Substance use, Behavioral intervention, Gender differences, South Africa

## Abstract

Little is known about gender effects of alcohol and drug use (AOD) among people living with HIV (PLWH) in resource-limited settings. Using multilevel models, we tested whether gender moderated the effect of *Khanya*, a cognitive-behavioral therapy-based intervention addressing antiretroviral (ART) adherence and AOD reduction. We enrolled 61 participants from HIV care and examined outcomes at 3- and 6-months compared to enhanced treatment as usual (ETAU). Gender significantly moderated the effect of *Khanya* on ART adherence (measured using electronically-monitored and biomarker-confirmed adherence), such that women in *Khanya* had significantly lower ART adherence compared to men in *Khanya*; no gender differences were found for AOD outcomes. Exploratory trajectory analyses showed men in *Khanya* and both genders in ETAU had significant reductions in at least one AOD outcome; women in *Khanya* did not. More research is needed to understand whether a gender lens can support behavioral interventions for PLWH with AOD.

*Trial registry* ClinicalTrials.gov identifier: NCT03529409. Trial registered on May 18, 2018.

**Supplementary Information:**

The online version contains supplementary material available at 10.1007/s10461-022-03765-8.

## Introduction

South Africa is home to the largest number of people living with HIV (PLWH) globally, approximately 7.9 million people [1]. Alcohol and other drug (AOD) use are highly prevalent among this population; approximately 30% of PLWH are estimated to have an alcohol use disorder as defined by the World Health Organization’s Alcohol Use Disorder Identification Test or the CAGE alcohol screening questionnaire [2] and about 13% of PLWH have problematic drug use [3]. Unhealthy AOD use, such as heavy episodic drinking or any injection drug use, can negatively affect engagement throughout the HIV care cascade, including worsening disease progression, reduced antiretroviral therapy (ART) adherence, decreased likelihood of viral load suppression, and increased likelihood of onward virus transmission [4–9]. Recent models suggest that AOD use may account for up to 40% of HIV-related deaths in South Africa (though the exact mechanisms of this association have not been elucidated) [10]. It is therefore important to address AOD use to improve HIV outcomes in South Africa.

An integrated intervention tested in Cape Town to address ART non-adherence and reduce AOD use (*Khanya*) showed that the intervention, a peer-delivered behavioral intervention based on behavioral activation, problem-solving therapy, motivational interviewing and mindfulness techniques [11, 12] led to significant improvements in ART adherence relative to enhanced treatment as usual (ETAU), a facilitated referral to a public, co-located AOD treatment program. Both groups showed improvements in severity, frequency, and quantity of AOD use [11]. However, given the substantial differences in the HIV and AOD epidemics by gender in South Africa and globally, important questions remain about how these findings may differ by gender.

In South Africa, the prevalence of HIV is almost twice as high in women than in men aged 15 to 49 years (26.3% in women versus 14.8% in men) [1]. However, once infected and aware of their status (which is > 90% among both men and women), men are generally less engaged than women throughout the HIV care cascade and are more likely to have an AIDS-related death [12–14]. With regard to AOD use, men aged 15 and older in the Western Cape province of South Africa are more likely than women to have any amount of AOD use within the past 3 months as measured by self-report and the alcohol biomarker phosphatidylethanol (PEth) [15–17], engage in binge drinking (defined as 5 or more drinks on a single occasion for both genders) [18, 19], more likely to meet the World Health Organization’s criteria for risky AOD use [20], and are more likely to be in AOD treatment [21]. In contrast, South African women face more stigma and discrimination in reporting AOD use as well as more barriers to AOD treatment engagement in general, including individual, interpersonal, community, and structural barriers, which may affect their recovery [22, 23].

Yet, few studies to date have adequately explored how gender may affect intervention outcomes for PLWH who use alcohol [e.g., 24]. A recent global review of alcohol interventions in PLWH found that over 60% of intervention studies almost exclusively enrolled men. Furthermore, very few studies have actively addressed both alcohol use and ART adherence (or other HIV outcomes) in the intervention [25]. There is a clear need to intentionally integrate gender into the design and analyses of interventions for this population to ensure interventions are effective for both genders. To address this gap, we conducted an exploratory study that aimed to examine whether the treatment effects observed in the *Khanya* behavioral intervention pilot trial [11] differed by gender. We evaluated the effect of gender on ART adherence, AOD use, severity, and quantity, and treatment utilization in both *Khanya* and ETAU groups.

## Methods

### Participants and Procedures

We recruited participants from an HIV clinic in Khayelitsha, a community with the highest HIV prevalence in the Western Cape province of South Africa [26]. All participants were PLWH on ART, between 18 and 65 years old, who scored in the moderate- or high-risk category for AOD use on the WHO Alcohol, Smoking and Substance Involvement Screening Test (ASSIST) [27] at screening, and were non-adherent to their ART, defined as any of the following: (a) missing an ART pharmacy refill in the past 3 months, (b) current unsuppressed viral load (≥ 400 copies/mL), or (c) re-initiation of first-line ART after a treatment gap or anyone on second-line ART. The study recruited both men and women using the same methods. Additional details on the study design, trial, and measurements can be found in the protocol and the main outcomes paper [11, 28].

Eligible and interested individuals completed a baseline assessment and returned to the clinic for a randomization visit. Participants were assigned 1:1 to *Khanya* versus ETAU. Participants randomized to *Khanya* received six sessions of a behavioral intervention for ART adherence and AOD use, based on Life Steps (a single-session problem-solving based intervention for ART adherence) [29], behavioral activation, and mindfulness (see [30] for a detailed description of the intervention). Participants randomized to ETAU received a facilitated referral to Matrix, an evidence-based co-located AOD treatment program [31]. Participants completed a post-treatment assessment approximately 3-months after the baseline visit and a follow-up visit approximately 6-months after the baseline assessment. All outcomes were assessed at baseline, post-treatment, and follow-up except for electronic ART adherence, which was not measured at follow-up. See primary outcomes paper for full study details [11]. The study was conducted in accordance with the Helsinki declaration and received ethics approval from the University of Cape Town and the City of Cape Town. All primary outcomes were registered a priori (ClinicalTrials.gov identifier: NCT03529409).

### Measures

#### HIV Outcomes

##### ART Adherence

Wisepill®, a real-time, wireless electronic adherence monitoring device [32] was used to measure ART adherence from baseline through 3-months (i.e., post-treatment). Participants were instructed to store their ART in the device, which transmits a real-time cellular signal when opened. Baseline adherence was measured as the percentage of days the device was opened ± 2 h of when the participant was supposed to take their medication over the 12 to 14 days prior to the study randomization visit. At 3-months, Wisepill® adherence was measured for seven days prior to the post-treatment assessment to capture the most recent adherence status. Since participants generally attended sessions weekly, using data only from the past seven days prior to post-treatment (rather than the past 14 days) likely gives the most accurate estimate of participant adherence after the intervention. Observations where the device battery was not functional were excluded (8.4% of total observations).

Dried blood spots (DBS) measuring concentration of tenofovir diphosphate (TFV-DP) in the blood provided an additional measure of adherence for participants on a tenofovir-based regimen (*n* = 44 first-line participants on tenofovir/emtricitabine/efavirenz; *n* = 1 second-line participant on tenofovir/emtricitabine/efavirenz, supplemented with two other HIV drugs).^1^ Continuous TFV-DP concentration (minimum quantification limit ≥ 16.6 fmol/punch) was used. TFV-DP values < 16.6 fmol/punch were coded as 0.

#### Substance Use Outcomes

##### Biomarker of Alcohol^2^

Phosphatidylethanol (PEth), which was analyzed from DBS, can detect alcohol use up to 21 days after consumption, and is highly correlated with quantity of alcohol consumed [33]. PEth testing was conducted at the US Drug Testing Laboratories. Continuous values of PEth (ng/mL) to measure alcohol use quantity were used in order to be consistent with the main outcomes of this trial [11].

##### AOD Severity and Problems

The Alcohol, Smoking and Substance Involvement Screening Test (ASSIST) is a self-report measure that assesses past 3-month AOD and related problems for alcohol, cannabis, cocaine, amphetamines, inhalants, sedatives, hallucinogens, opioids, and other drug involvement. It has previously been validated for use in the South African context [27]. We used the defined risk categories for alcohol and drug use: low = 0–10 (alcohol) or 0–3 (drugs); moderate = 11–26 (alcohol) or 4–26 (drugs); high ≥ 27 (both alcohol and drugs).

##### AOD Quantity

The timeline followback (TLFB), a calendar method used to aid in the recall of substance use [34], was used to assess quantity of alcohol (number of standard drinks) in the past two weeks. We used empty, locally recognizable alcohol containers to aid in recall.

#### Treatment Utilization

##### ETAU Treatment Utilization

For ETAU participants, the enhancement to standard of care was a facilitated referral to Matrix, with the focus on uptake of the referral. The majority of participants (~ 70%) who attended Matrix only attended one session [11]. As a result, we examined the percent of the ETAU treatment arm who used the referral and attended at least one Matrix session as our definition of treatment utilization.

##### Khanya Treatment Utilization

All participants randomized to *Khanya* attended at least one session of the intervention (as reported in the primary outcomes paper [11]). The intervention was comprised of six regular treatment sessions plus up to six additional boosters. We therefore examined the total number of sessions attended (possible range 0–12) as our indicator of utilization.

### Data Analytic Plan

Our primary analyses compared mean differences in the outcome variables (e.g., Wisepill®, ASSIST score) between gender and treatment arms across all post-randomization timepoints (i.e., combining the 3- and 6-months timepoints), controlling for baseline level of the outcome variable. Both post-treatment and follow-up data were included in the same model for all outcome variables, except for Wisepill®, which was only assessed through post-treatment. The models accounted for nesting within participant using multilevel modeling [35]. Analyses used an intent-to-treat framework [36], which included all available data, and missing data were treated as missing at random. We did not include additional control variables in the model due to the exploratory nature of the analyses. All analyses were run using SAS version 9.4.

To supplement this primary analytic approach, we also assessed change in outcomes over time by gender and treatment arm to evaluate distinct trajectories for each group over time. Time was treated categorically to capture differing rates of change between the major time points. Gender was included as a predictor of model intercept and slopes, with the primary estimates of interest being the Gender × Treatment group and Time × Gender × Treatment group interactions. All models included a random intercept. PROC MIXED was used to model TFV-DP and PEth, which were both continuous variables. Percentage of days adherent to ART was modeled as a continuous variable using PROC REG for the primary analysis at post-treatment and PROC GLIMMIX for the secondary analysis from baseline to post-treatment.^3^ PROC GLIMMIX was used to model the ASSIST using the cumulative logit link and number of drinks on the TLFB using the log link for both primary and secondary analyses. Significant interactions (*p* < 0.05) were graphed based on model-implied means and probed as appropriate using post-hoc tests from the LSMEANS function. The significance level for probed interactions was also set at *p* < 0.05 given the exploratory nature of the study.

## Results

Table 1 presents the demographic and clinical characteristics by gender and treatment condition. A total of *N* = 61 participants were enrolled in the study. All participants identified as binary “female” or “male,” though gender options of “transmale” and “transfemale” were provided to participants. Fifty-five percent of the sample were women (*n* = 33), with *n* = 13 randomized to *Khanya* and *n* = 20 to ETAU. Men comprised 45% of the sample (*n* = 28), with *n* = 17 randomized to *Khanya* and *n* = 11 to ETAU. Results suggest several demographic and clinical differences between men and women in the two treatment conditions, though these were not evaluated statistically. Women, as compared to men, had lower levels of employment and had better control of their HIV, as measured by viral suppression, CD4 count, and second-line treatment status. Women also had less alcohol consumption, as measured by PEth.^4^ Table 1 presents descriptive data for all outcome measures at each time point by gender. Results of the primary analyses (post-randomization timepoint comparisons, controlling for baseline values) are presented in Tables 2 and 3. Results of the relevant interactions for the secondary analyses of trajectories (Time x Gender; Time x Gender x Treatment group) for each outcome are presented in Table 4. Full model results for trajectory analyses are presented as supplemental tables. Results are detailed below.

**Table 1:** Demographic and clinical characteristics of the sample by gender and treatment group

Characteristic	Men			Women		
	Total (*N* = 28)	Khanya (*n* = 17)	ETAU (*n* = 11)	Total (*N* = 33)	Khanya (*n* = 13)	ETAU (*n* = 20)
Age, *M (SD)*	40.5 (9.0)	41.6 (9.7)	38.7 (7.9)	34.0 (9.3)	37.4 (11.3)	31.9 (7.1)
% graduated high school or above (n)	25.0 (7)	35.3 (6)	9.1 (1)	21.2 (7)	15.4 (2)	25.0 (5)
% casual or full-time employment (n)	28.6 (8)	17.7 (3)	45.5 (5)	15.2 (5)	7.7 (1)	20.0 (4)
% married or common-law (n)	28.6 (8)	11.8 (2)	54.6 (6)	24.2 (8)	7.7 (1)	35.0 (7)
HIV characteristics						
Years since HIV diagnosis, *M (SD)*	6.1 (3.0)	6.0 (2.9)	6.2 (3.3)	6.4 (6.1)	8.7 (8.4)	5.0 (3.5)
% suppressed viral load (n)^**§**^	53.6 (15)	41.2 (7)	72.7 (8)	72.7 (24)	61.5 (8)	80.0 (16)
CD4 count	280 (212)	258 (169)	313 (271)	458 (259)	424 (227)	479 (281)
% on second-line (n)	35.7 (10)	41.2 (7)	27.3 (3)	18.8 (6)^i^	0 (0)	30.0 (6)

Outcome measures	Baseline (*N* = 28)	3-month (*N* = 23)	6-month (*N* = 25)	Baseline (*N* = 33)	3-month (*N* = 29)	6-month (*N* = 31)
% days adherent Wisepill®, *M (SD)*	47.8% (33.2)	54.4% (38.2)^c^	–	54.5% (28.6)^i^	34.8% (35.9)^d^	–
ART concentration (fmol 3 mm/punch)^h^	1162 (517)	1285 (805)^f^	709 (522)^g^	1000 (509)	856 (480)^b^	648 (411)^b^
PEth score, *M SD*	821 (680)***	554 (425)^a^	668 (579)^b^	355 (409)***	372 (352)	287 (285)^e^
% moderate or high risk on ASSIST	100% (28)	91.3% (21)	80.0% (20)	100% (33)	93.1% (27)	83.9 (26)
Average drinks on TLFB, *M SD*	7.29 (4.10)	4.70 (4.24)	4.44 (2.89)	7.88 (5.33) ^*j*^	6.07 (3.31) ^*j*^	5.03 (4.36)

Comparison of means in the outcome measures are between gender at each time point

*ETAU* enhanced treatment as usual, *ART* antiretroviral therapy, *PEth* phosphatidylethanol, *ASSIST* Alcohol, Smoking and Substance Involvement Screening Test, *TLFB* timeline followback

***p < 0.001

^**^**^Data from randomization visit

^**^^**^Data from screening visit

^**§**^Viral suppression defined as < 400 copies/mL

^a^*n* = 22

^b^*n* = 24

^c^*n* = 27

^d^*n* = 28

^e^*n* = 30

^f^*n* = 13

^g^*n* = 11

^h^Participants on TDF-based regimen only, *n* = 18 men and *n* = 25 women at baseline

^i^One participant was not actively taking ART at the time of study enrolment so *n* = 32

^j^One participant was enrolled in the study for drugs and did not complete TLFB for alcohol at this timepoint

**Table 2:** Gender interaction effects for adherence outcomes in models predicting post-randomization timepoints

	Wisepill®			TFV-DP		
	B (SE)	t	p	B (SE)	t	p
Baseline value of outcome	0.32 (0.15)	2.10	0.04	0.49 (0.14)	3.53	0.001
Intercept (men in Khanya)	0.57 (0.11)	5.36	< 0.001	676 (199)	3.40	0.001
Gender effect (women in Khanya)	− 0.29 (0.13)	− 2.20	0.03	− 473 (204)	− 2.31	0.02
Treatment effect (men in ETAU)	− 0.44 (0.13)	− 3.42	0.001	− 414 (226)	− 1.83	0.07
Gender x Treatment group (women in ETAU)	0.26 (0.18)	1.40	0.16	441 (283)	1.56	0.12

*TFV-DP* Tenofovir diphosphate, *ETAU* enhanced treatment as usual

**Table 3:** Gender effects for substance use outcomes in models predicting post-randomization timepoints

	PEth			TLFB, *M* drinks			ASSIST		
	B (SE)	t	p	B (SE)	t	p	B (SE)	t	p
Baseline value of outcome	.48 (.07)	7.08	< 0.001	0.03 (0.02)	1.48	0.14	2.03 (0.64)	3.19	0.002
Intercept (men in Khanya)	191 (98)	1.95	0.05	1.11 (0.27)	4.11	< 0.001	Moderate or high risk group: 1.65 (0.67)	2.47	0.01
							High risk group only: − 2.00 (0.71)	− 2.82	0.006
Gender effect (women in Khanya)	− 57 (116)	− 0.49	0.62	0.17 (0.28)	0.59	0.56	0.49 (0.79)	0.63	0.53
Treatment effect (men in ETAU)	− 1 (116)	− 0.01	0.99	− 0.13 (0.31)	− 0.42	0.67	− 0.08 (0.84)	− 0.09	0.92
Gender × treatment group (women in ETAU)	15 (155)	0.09	0.92	0.18 (0.42)	0.41	0.68	− 0.52 (1.13)	− 0.46	0.64

*PEth* phosphatidylethanol, *TLFB* timeline followback, *ASSIST* Alcohol, Smoking and Substance Use Involvement Test, *ETAU* enhanced treatment as usual

**Table 4:** Gender interaction effects for adherence and substance use outcomes for trajectory analyses

Effect	Time × gender			Time × gender × treatment group		
	DF	*F* or *t*	*p*	DF	*F or t*	*p*
Wisepill®	1, 50	− 3.19	0.002	1, 50	2.27	0.02
Tenofovir diphosphate (TFV-DP)	2, 62	1.59	0.21	2, 62	0.86	0.42
Phosphatidylethanol (PEth)	2, 97	3.62	0.03	2, 97	1.09	0.33
ASSIST risk category	2, 100	.34	0.71	2, 100	1.27	0.28
Average number of drinks, TLFB	2, 98	1.32	0.27	2, 98	3.57	0.03

Wisepill® results are calculated using a *t* test because there are only two time points. All others are *F* tests

*ASSIST* Alcohol, Smoking and Substance Use Involvement Test, *TLFB* Timeline Followback

### Adherence Outcomes

The primary analyses for Wisepill® adherence showed significantly lower post-treatment adherence in ETAU men (*B* = − 0.44, *p* = 0.001) and *Khanya* women (*B* = − 0.29, *p* = 0.03), as compared to *Khanya* men. The Gender × Treatment group interaction was not significant (*B* = 0.26, *p* = 0.16), which indicates that adherence among women in ETAU was not significantly different than adherence among men in ETAU or women in *Khanya.* The primary analyses for TFV-DP mirrored these findings, with men in ETAU (*B* = − 414, *p* = 0.07) and women in *Khanya* (*B* = − 473, *p* = 0.02) having lower concentration of ART in the blood than men in *Khanya*.

**Fig. 1:**
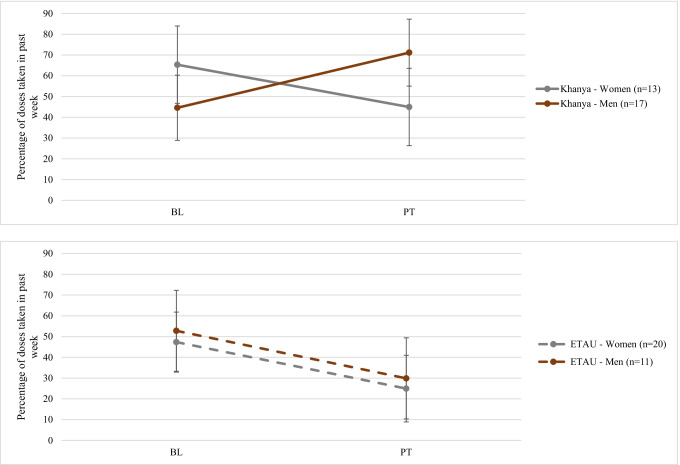
Interaction between gender, time, and treatment group for Wisepill® adherence

The trajectory analyses further highlight these differences. The three-way interaction of Time × Gender × Treatment group for Wisepill® (*p* = 0.02) shows that men in *Khanya* improved their ART adherence over the course of the intervention, whereas adherence worsened for women in *Khanya* between baseline and post-treatment. This declining pattern was also seen for both men and women in ETAU (see Fig. 1). The model-implied estimates suggest that men in *Khanya* increased their adherence by approximately 26 percentage points, whereas all other groups decreased their adherence by 20 to 23 percentage points. Although the gender interactions were not significant in the trajectory model predicting concentration of TFV-DP (*ps* > 0.05), a line graph of the data (Fig. S1) shows that men in *Khanya* demonstrated an increase in TVF-DP concentration during the active intervention from baseline to post-treatment, though not to follow-up; all other groups showed declines in this outcome. See Table S1 for Wisepill® and TFV-DP trajectory analyses.

### AOD Outcomes

**Fig. 2:**
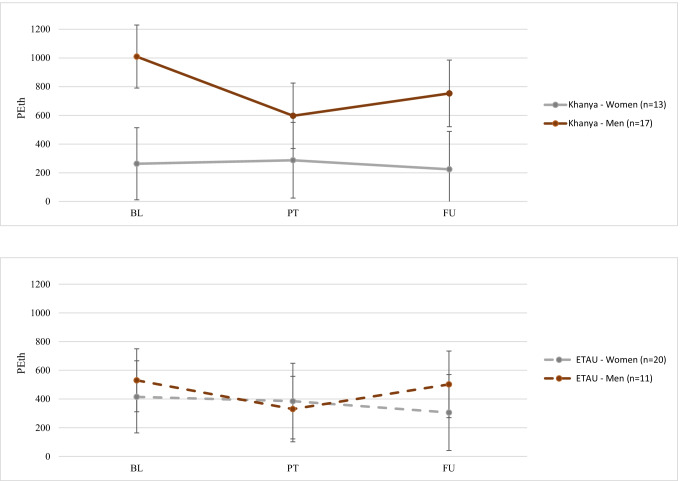
Interaction between gender, time, and treatment group for continuous PEth score

The primary analyses for PEth, TLFB average number of drinks, and ASSIST scores do not show any significant differences by gender and treatment group in the outcomes combined across the 3- and 6-months timepoints (*p*s > 0.05, see Table 3), after accounting for baseline level of the outcome variable. The trajectory analyses provide some additional data on how these outcomes change over time by group. There was a significant Time × Gender interaction (*p* = 0.03) in the trajectory model predicting PEth, but not a significant three-way interaction for Time × Gender × Treatment group (*p* = 0.33). Figure 2 presents a graphical depiction of the interaction. Men in both *Khanya* and ETAU showed significant reductions in PEth from baseline to post-treatment (mean difference = − 306 ng/mL, *t* = − 3.48, *p* < 0.001), but this effect was reduced at follow-up (mean difference = − 142 ng/mL, *t* = − 1.74, *p* = 0.08). However, women did not show significant reductions in PEth over time, either at post-treatment (mean difference = − 3 ng/mL, *t* = − 0.04, *p* = 0.96) or follow-up (mean difference = − 74 ng/mL, *t* = − 0.99, *p* = 0.32), across both treatment conditions.

**Fig. 3:**
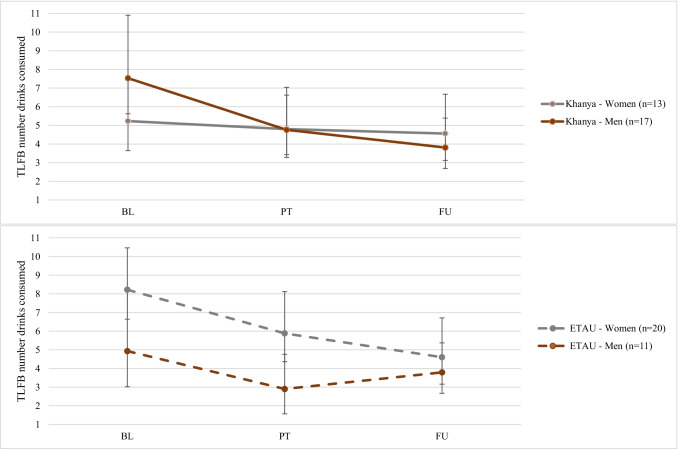
Interaction between gender, time, and treatment group for average number of drinks consumed on days drinking

We observed a significant three-way interaction of Time × Gender × Treatment group (*p* = 0.03) in the trajectory model predicting average drinks consumed on days drinking on the TLFB, presented in Fig. 3. For *Khanya* men, there was a significant reduction in the average number of drinks from baseline to post-treatment (log odds mean difference = − 0.46, *t* = − 3.27, *p* = 0.001) and at follow-up (log odds mean difference = − 0.68, *t* = − 4.54, *p* < 0.001). Men in *Khanya* went from an average of 7.5 drinks at baseline to 4.8 drinks at post-treatment and 3.8 drinks at follow-up. For ETAU men, there was a significant reduction in average number of drinks at post-treatment only (log odds mean difference = − 0.53, *t* = − 2.22, *p* = 0.02). Women in ETAU showed similar decreases in the average number of drinks from baseline to post-treatment (log odds mean difference = − 0.33, *t* = − 2.73, *p* = 0.007), which remained significant at follow-up (log odds mean difference = − 0.58, *t* = − 4.58, *p* < 0.001). The average number of drinks remained stable for women in *Khanya* at both post-treatment (log odds mean difference = − 0.08, *t* = − 0.47, *p* = 0.63) and follow-up (log odds mean difference = − 0.14, *t* = − 0.77, *p* = 0.44), as compared to baseline.

No significant interaction effects with gender were observed in the model predicting the trajectory of AOD risk categories based on the ASSIST (*p*s > 0.05). Visually, graphing the ASSIST results shows a consistent picture where women in *Khanya* were the only group to show an increased probability of being in the high-risk AOD category during the follow-up period (see Fig. S2). However, this outcome was not statistically significant.

### Treatment Utilization

Finally, we examined intervention session utilization by gender for both participants randomized to *Khanya* and to ETAU. For *Khanya*, we found that men attended an average of 6.24 sessions (regular sessions plus boosters) (*SD* = 1.86), compared to 4.54 (*SD* = 2.33) for women, which was a significant difference (*p* = 0.03). For ETAU, we found that 72.7% (8/11) of men attended Matrix at least once, compared to 85.0% of women (17/20). However, this difference was not significant (*p* = 0.63).

## Discussion

The goal of the current study was to conduct an exploratory investigation of whether men and women showed differential improvements in ART adherence and AOD use in South Africa after receiving a behavioral intervention. Although preliminary, this is an important contribution to the field given lack of research evaluating gender differences in the effectiveness of interventions that address the intersection of these two epidemics [25]. Our primary and secondary analyses showed that men who received the *Khanya* intervention had significantly higher ART adherence at 3- and 6-months, using Wisepill® real-time electronic adherence monitoring and TFV-DP, in comparison to women in *Khanya* and men in ETAU. The comparison of mean differences by gender and treatment arm in AOD outcomes, combined across the 3- and 6-month timepoints, did not show any differences. Yet, the trajectory analyses for AOD, which modeled discrete changes over time using baseline, 3-months, and 6-months timepoints, showed that across both treatment conditions, men reduced their alcohol use more than women based on the biomarker-confirmed alcohol use outcome, PEth, and that women in *Khanya* appeared to experience the least improvement across all outcomes examined. Finally, men in the *Khanya* treatment arm attended a greater number of sessions than women, but gender differences in ETAU attendance were not observed. Findings shed light on the possibility of recognizing gender differences in response to combined interventions for ART and AOD.

In sub-Saharan Africa, men’s rate of engagement throughout the HIV care cascade is lower than women’s, including lower ART initiation and adherence [37–39]. Despite this, we observed a pattern of adherence where women who received the *Khanya* intervention changed in similar ways to the men and women of ETAU, namely declining adherence over time, which resulted in significantly lower post-treatment Wisepill® adherence than men in *Khanya*. This finding was further supported by the TFV-DP results. Although descriptively it appeared that women had more controlled HIV at baseline than men, given the percent with a suppressed viral load and CD4 count, there were no significant gender differences in the adherence outcomes at baseline, indicating that a ceiling effect for women was not a concern.

A meta-analysis of interventions used to improve ART adherence for women found that very few studies actually adapted their interventions to address gender dynamics or gender empowerment [40]. Given that the HIV burden is almost twice as high in women than men in South Africa [1], it is necessary to understand what treatment approaches work to successfully engage women into care and what intervention components actually help women better adhere to their ART. HIV stigma, potential violence by partners upon HIV status disclosure, and histories of violence victimization may be some of the factors affecting women’s use of Wisepill® and their ART adherence [41–44].

With regard to AOD outcomes, we did not find any evidence of mean differences in outcomes by gender and treatment groups, when combining across post-treatment and follow-up timepoints. Yet, when we examined trajectories of change, we observed a reduction in men’s biomarker-confirmed alcohol use, as compared to women, across both treatment conditions. Both groups of men showed significant reductions in PEth from baseline to post-treatment, though PEth scores again increased at follow-up. An important consideration is that men had heavier alcohol use at baseline than women, which makes it possible that results are due to regression to the mean. This would also explain why, after accounting for baseline severity of PEth, there were no gender differences in the primary analysis. However, women in both treatment groups were still demonstrating levels of unhealthy drinking well beyond the cutoff of ≥ 50 ng/mL at baseline (average of 355 ng/mL), leaving ample room for improvement. Taken together, it may be that these AOD interventions are more helpful for reducing very high levels of alcohol consumption, or they are more responsive to the needs of men. This cannot be determined from the current study and will be an important future direction from this work.

The study findings should be considered in light of factors that are known to influence adherence and AOD behaviors for each gender. This includes women being more likely than men to use AOD as a coping strategy in response to trauma [45–48] and the role of masculinity in promoting heavy AOD use and reducing HIV care participation [38, 49–51]. Interventions should potentially consider such factors and test whether including them in treatment further improves outcomes.

This study has several strengths and limitations that should be acknowledged. Study strengths include the use of a randomized, longitudinal design, validated assessment tools, and treatment implemented in a real-world clinical setting. We were also able to recruit similar numbers of women and men into the study (55% women), an advancement over many previous alcohol studies for PLWH that almost exclusively recruited men [25]. Furthermore, the fact that 45% of the sample were men is considerably higher than the percentage of men typically observed in local ART clinics (~ 35% men) [52]. This may suggest that focusing on AOD in the context of HIV care may be a strategy for engaging men in treatment, which should be evaluated in future work.

A primary limitation of this study is the fact that this was a pilot study (small sample, especially for TFV-DP, and short follow-up period) used to investigate the possibility of gender effects, which limits our ability to detect significant differences as well as the ability to assess how men and women fared over the longer-term. However, the fact that we found several significant differences with consistent findings, including three-way interactions in our trajectory analyses, reduces concern about spurious findings or being underpowered to detect effects. A second limitation is that we did not stratify participants based on gender or evaluate theoretically relevant mechanisms to explain the potential gender differences that were observed. Future studies should stratify randomization by gender and be powered to examine differences in treatment outcomes by gender, including over the longer-term. Moreover, in this study men had significantly higher scores on PEth at baseline than women, indicating the possibility that in the trajectory analyses, the greater changes observed in men may be due to regression to the mean. That said, there were no baseline differences by gender in the other AOD and HIV outcomes in which men demonstrated greater improvements than women, reducing the concern that all results are due to regression to the mean.

Overall, this exploratory study showed the possibility that men and women fare differently after receiving an intervention targeting ART adherence and AOD use. Across all outcomes, men who received the *Khanya* intervention appeared to experience the most gains compared to women in *Khanya*, and a similar gender pattern was observed in the comparison group of the enhanced referral to the co-located AOD treatment program. Adding gender-relevant components to the intervention and addressing gender-specific barriers to treatment utilization may be important next steps to evaluate whether this can improve treatment outcomes for AOD and ART adherence for both women and men.

## Supplementary Information

Below is the link to the electronic supplementary material.

Supplementary figures1 (DOCX 28 kb)

Supplementary tables (DOCX 24 kb)

## Data Availability

Data are available upon request of the first author.

## Footnotes

1 Data were missing for two second-line participants who were on a tenofovir-based regimen but for whom TFV-DP testing was not conducted at any timepoint.

2 The primary outcomes paper included urinalysis to assess alcohol and drug use. However, the results showed no significant changes over time across either *Khanya* or ETAU groups, so was not included in this analysis.

3 When predicting trajectories or change over time, as in the secondary analysis, at least three timepoints are needed for PROC MIXED, which is why PROC GLIMMIX was chosen instead.

4 Given some potential differences between groups, sensitivity analyses were run controlling for baseline employment and viral suppression status. Substantive findings did not change. Results can be obtained from the first author.

## Code Availability

Code is available upon request of the first author.
