# Supplementary material for: Gender Moderates Results of a Randomized Clinical Trial for the Khanya Intervention for Substance Use and ART Adherence in HIV Care in South Africa
Source: AIDS Behav. 2022 Jul 27;26(11):3630–41. doi: 10.1007/s10461-022-03765-8 (PMC9550692; doi:10.1007/s10461-022-03765-8)
Supplement: Supplementary file 1 — Supplementary figures1 (DOCX 28 kb) [file 10461_2022_3765_MOESM1_ESM.docx]

*Figure S1.* Non-significant Interaction between Gender, Time, and Treatment Group for Tenofovir Diphosphate Concentration in Dried Blood Spots for Patients on Tenofovir-based ART Regimen.

*Figure S2.* Non-significant Interaction between Gender, Time, and Treatment Group for Probability of being in the High-Risk Category on the WHO-ASSIST.
