# Supplementary material for: Gender Moderates Results of a Randomized Clinical Trial for the Khanya Intervention for Substance Use and ART Adherence in HIV Care in South Africa
Source: AIDS Behav. 2022 Jul 27;26(11):3630–41. doi: 10.1007/s10461-022-03765-8 (PMC9550692; doi:10.1007/s10461-022-03765-8)
Supplement: Supplementary file 2 — Supplementary tables (DOCX 24 kb) [file 10461_2022_3765_MOESM2_ESM.docx]

Table S1

*Models Predicting Wisepill^®^ Adherence and Tenofovir Diphosphate Concentration over Time by Gender and Treatment Group*

|  | Wisepill^®^ | | | | Tenofovir Diphosphate (TFV-DP) | | | |
| --- | --- | --- | --- | --- | --- | --- | --- | --- |
| Effect | Estimate (SE) | 95% CI | *t* | *p* | Estimate (SE) or DF | 95% CI | *F* or *t* | *p* |
| Intercept [men in Khanya at BL] | .446 (.078) | [.290, .603] | 5.71 | <.001 | 1068 (171) | [722, 1414] | 6.23 | <.001 |
| Gender effect [women in Khanya at BL] (intercept) | .207 (.121) | [-.035, .450] | 1.71 | .09 | -9 (239) | [-492, 473] | -.04 | .97 |
| Treatment effect [men in ETAU at BL] (intercept) | .082 (.125) | [-.168, .332] | .66 | .51 | 213 (257) | [-306, 731] | .83 | .41 |
| Gender x Treatment group effect [women in ETAU at BL] (intercept) | -.262 (.171) | [-.605, .081] | -1.53 | .13 | -259 (337) | [-939, 421] | -.77 | .44 |
| Time^*^ | 1, 50 | -- | -- | -- | 2, 62 | -- | 10.08 | <.001 |
| PT for men in Khanya | .270 (.095) | [.074, .457] | 2.79 | .007 | 335 (185) | [-35, 705] | 1.81 | .07 |
| FU for men in Khanya | -- | -- | -- | -- | -243 (222) | [-686, 199] | -1.10 | .27 |
| Time x Treatment group^*^ | 1, 50 | -- | -- | -- | 2, 62 | -- | 1.07 | .34 |
| PT for men in ETAU | -.495 (.150) | [-.796, -.193] | -3.30 | .001 | -442 (294) | [-1030, 145] | -1.50 | .13 |
| FU for men in ETAU | -- | -- | -- | -- | -366 (308) | [-981, 249] | -1.19 | .23 |
| Time x Gender^*^ | 1, 50 | -- | -- | -- | 2, 62 | -- | 1.59 | .21 |
| PT for women in Khanya | -.469 (.147) | [-.765, -.174] | -3.19 | .002 | -515 (261) | [-1037, 8] | -1.97 | .05 |
| FU for women in Khanya | -- | -- | -- | -- | -42 (284) | [-609, 526] | -.15 | .88 |
| Time x Gender x Treatment group^*^ | 1, 50 | -- | -- | -- | 2, 62 | -- | .86 | .42 |
| PT for women in ETAU | .475 (.209) | [.055, .895] | 2.27 | .02 | 491 (374) | [-257, 1238] | 1.31 | .19 |
| FU for women in ETAU | -- | -- | -- | -- | 206 (383) | [-559, 971] | .54 | .59 |

*Note.* BL = baseline. PT = post-treatment. FU = follow-up. ETAU = enhanced treatment as usual. ^*^The Wisepill^®^ *t* test statistic and *p* value for the interactions are identical to the individual effect of the interaction.

Table S2

*Models Predicting PEth and Timeline Followback over Time by Gender and Treatment Group*

|  | PEth | | | | TLFB number of drinks (log odds) | | | |
| --- | --- | --- | --- | --- | --- | --- | --- | --- |
| Effect | Estimate (SE)  or DF | 95% CI | F or t | p | Estimate (SE)  or DF | 95% CI | F or t | p |
| Intercept [men in Khanya at BL] | 1009 (111) | [788, 1231] | 9.12 | <.001 | 2.02 (.15) | [1.72, 2.31] | 13.70 | <.001 |
| Gender effect [women in Khanya at BL] (intercept) | -746 (168) | [-1083, -410] | -4.44 | <.001 | -.37 (.23) | [-.83, .10] | -1.57 | .12 |
| Treatment effect [men in ETAU at BL] (intercept) | -479 (177) | [-833, -126] | -2.71 | .008 | -.42 (.25) | [-.92, .07] | -1.72 | .09 |
| Gender x Treatment group effect [women in ETAU at BL] (intercept) | 631 (240) | [151, 1112] | 2.63 | .01 | .88 (.33) | [.21, 1.55] | 2.63 | .01 |
| Time^§^ | 2, 97 | -- | 3.91 | .02 | 2, 98 | -- | 15.31 | <.001 |
| PT for men in Khanya | -412 (102) | [-615, -210] | -4.04 | <.001 | -.46 (.14) | [-.74, -.18] | -3.27 | .001 |
| FU for men in Khanya | -256 (105) | [-464, -49] | -2.45 | .01 | -.68 (.15) | [-.98, -.38] | -4.54 | <.001 |
| Time x Treatment group | 2, 97 | -- | .33 | .71 | 2, 98 | -- | .46 | .63 |
| PT for men ETAU | 213 (176) | [-137, 562] | 1.21 | .23 | -.07 (.28) | [-.62, .48] | -.26 | .79 |
| FU for men ETAU | 228 (164) | [-96, 553] | 1.40 | .16 | .42 (.25) | [-.07, .91] | 1.70 | .09 |
| Time x Gender | 2, 97 | -- | 3.62 | .03 | 2, 98 | -- | 1.32 | .27 |
| PT for women in Khanya | 437 (156) | [126, 747] | 2.79 | .006 | .37 (.23) | [-.08, .82] | 1.65 | .10 |
| FU for women in Khanya | 217 (158) | [-96, 531] | 1.38 | .17 | .55 (.23) | [.09, 1.01] | 2.35 | .02 |
| Time x Gender x Treatment group | 2, 97 | -- | 1.09 | .33 | 2, 98 | -- | 3.57 | .03 |
| PT for women in ETAU | -268 (232) | [-728, 193] | -1.15 | .25 | -.18 (.35) | [-.88, .52] | -.51 | .61 |
| FU for women in ETAU | -299 (222) | [-739, 141] | -1.35 | .18 | -.87 (.33) | [-1.52, .21] | -2.62 | .01 |

*Note.* BL = baseline. PT = post-treatment. FU = follow-up. ETAU = enhanced treatment as usual. PEth = Phosphatidylethanol. TLFB = Timeline Followback.

Table S3

*Model Predicting WHO-ASSIST Moderate and High Risk Categories over Time by Gender and Treatment Group*

| Effect | Estimate (SE) or DF | 95% CI | *F* or *t* | *p* |
| --- | --- | --- | --- | --- |
| Intercept (moderate or high risk group) | 4.99 (.96) | [3.07, 6.90] | 5.21 | <.001 |
| Intercept effect (high risk group) | .68 (.75) | [-.82, 2.18] | .91 | .36 |
| Gender effect [women in Khanya at BL] (intercept) | .28 (1.16) | [-2.03, 2.60] | .24 | .80 |
| Treatment effect [men in ETAU at BL] (intercept) | .39 (1.23) | [-2.06, 2.85] | .32 | .75 |
| Gender x Treatment group effect [women in ETAU at BL] (intercept) | -1.67 (1.66) | [-4.99, 1.66] | -1.01 | .31 |
| Time^§^ | 2, 100 | -- | 6.54 | .002 |
| PT for men in Khanya | -1.07 (.87) | [-2.79, .66] | -1.22 | .22 |
| FU for men in Khanya | -2.25 (.88) | [-4.00, -.50] | -2.55 | .01 |
| Time x Treatment group | 2, 100 | -- | .06 | .93 |
| PT for men in ETAU | -1.03 (1.45) | [-3.90, 1.84] | -.71 | .47 |
| FU for men ETAU | .20 (1.40) | [-2.57, 2.98] | .15 | .88 |
| Time x Gender | 2, 100 | -- | .34 | .71 |
| PT for women in Khanya | -.65 (1.32) | [-3.27, 1.97] | -.49 | .62 |
| FU for women in Khanya | 1.26 (1.30) | [-1.32, 3.85] | .97 | .33 |
| Time x Gender x Treatment group | 2, 100 | -- | 1.27 | .28 |
| PT for women in ETAU | 2.01 (1.91) | [-1.78, 5.81] | 1.05 | .29 |
| FU for women in ETAU | -.99 (1.86) | [-4.68, 2.69] | -.54 | .59 |

*Note.* BL = baseline. PT = post-treatment. FU = follow-up. ETAU = enhanced treatment as usual. *values are ordered as baseline, post-treatment, and follow-up.
